# Efficacy and safety of *Brucea javanica* oil emulsion for liver cancer: A protocol for systematic review and meta-analysis

**DOI:** 10.1097/MD.0000000000023197

**Published:** 2020-11-20

**Authors:** Didi Luo, Daorui Hou, Tiancheng Wen, Meiling Feng, Haiming Zhang

**Affiliations:** a Department of Oncology; b Department of Traditional Chinese Medicine Oncology, The First People's Hospital of Xiangtan City, Xiangtan, Hunan Province, China.

**Keywords:** *Brucea javanica* oil emulsion, liver cancer, protocol, systematic review and meta-analysis

## Abstract

**Background:**

*Brucea javanica* oil emulsion (BJOE), extracted from the Chinese herb *Bruceae Fructus (Yadanzi)*, is a broad-spectrum anti-tumor drug and has been widely used for the treatment of liver cancer in China. The aim of this study is to systematically investigate the efficacy and safety of BJOE for the treatment of liver cancer.

**Methods:**

Seven electronic databases including the Cochrane Library, PubMed, Excerpt Medica Database, Chinese Biomedical Literature Database, China National Knowledge Infrastructure, China Scientific Journal Database, and Wanfang Database will be systematically retrieved for data extraction from their inceptions to September 2020. Cochrane Risk of Bias tool will be used to assess the risk of bias of included studies. The RevMan 5.4 and Stata 16.0 software will be applied for statistical analyses. Statistical heterogeneity will be computed by *I*^*2*^ tests. Sensitivity analysis will be conducted to evaluate the stability of the results. The publication bias will be evaluated by funnel plots and Egger test. The quality of evidence will be assessed by the GRADE system.

**Results:**

The results of our research will be published in a peer-reviewed journal or presenting the findings at a relevant conference.

**Conclusion:**

The conclusion of this study will provide helpful evidence of the effect and safety of BJOE for the treatment of liver cancer in clinical practice.

**OSF registration number:**

10.17605/OSF.IO/UC8XQ

## 1 Introduction

Liver cancer is one of the most common malignancies and the second most common cause of cancer-related death worldwide.^[1]^ While there have been some developments in advancing therapeutic options in this disease, these have admittedly been modest to date.^[2]^ The increase in liver cancer incidence, the undruggable nature of liver cancer mutations, and unresponsiveness of these tumors to therapy highlight the urgency required for more effective and safer treatments.^[3–5]^

Traditional Chinese medicine (TCM) has been effectively applied in treating malignant diseases for thousands of years.^[6,7]^ In recent years, researchers have found that many TCMs, as well as compounds extracted from certain Chinese medicines, have outstanding anti-tumor effect.^[8–10]^ As one of the famous TCM preparations, *Brucea javanica* oil emulsion (BJOE) is extracted from the Chinese herb *Bruceae Fructus (Yadanzi)*, and has been often employed as adjunctive therapy for the treatment of various cancers including lung cancer,^[11]^ gastric cancer,^[12]^ esophageal cancer,^[13]^ and liver cancer.^[14,15]^

Studies have shown that the mechanisms of antitumor activity of BJOE may include inhibiting DNA polymerase activity,^[16]^ arresting the tumor cell division cycle,^[17]^ disrupting the cellular energy metabolism, and depressing the expression of vascular endothelial growth factor.^[11]^ Other studies suggest that BJOE can effectively reverse the multidrug resistance of tumor cells^[18]^ and increase the sensitivity of cancer cells to chemotherapy and radiotherapy.^[11,19]^ A pooled result of a meta-analysis showed that BJOE was more favorable for 1-year and 2-year survival rate than other TCM preparations for the patients with liver cancer.^[15]^

Many studies have proved that BJOE can perform a synergetic antitumor effect by improving tumor response, improving the quality of life, and reducing the incidence of adverse events during radiochemotherapy.^[20–22]^ But its clinical efficacy for liver cancer is still not systematically evaluated. Thus, we prepare to perform this meta-analysis to investigate the clinical efficacy and safety of BJOE in the treatment of liver cancer, which may provide a scientific reference for clinical application.

## 2 Methods and analysis

The protocol of our meta-analysis will be carried out under the guideline of the Preferred Reporting Items for Systematic Review and Meta-Analysis Protocols (PRISMA-P) recommendations.^[23]^ This work was prospectively registered at Open Science Framework (https://osf.io/uc8xq) with a DOI: 10.17605/OSF.IO/UC8XQ.

### 2.1 Inclusion criteria

#### 2.1.1 Type of study

All randomized controlled trials (RCTs) that investigated the efficacy and safety of BJOE for the treatment of liver cancer will be included in this systematic review without language restriction. Nonrandomized control studies, qualitative studies, laboratory studies, and observational study will be excluded in the review.

#### 2.1.2 Types of participants

Any participants who are diagnosed as liver cancer will be considered for inclusion. There are no limits to research subjects’ age, gender, race, condition duration, or intensity.

#### 2.1.3 Types of interventions

Interventions to be reviewed are BJOE alone or combinations with other interventions to treat liver cancer. When BJOE used as combinations with other treatments, the control group should also receive the same combination of treatments.

#### 2.1.4 Types of outcomes

The primary outcomes in present analysis included overall survival and progression-free survival. The secondary outcomes included overall response rate, disease control rate, quality of life improved rate, and adverse events.

### 2.2 Search strategy

To ascertain the relevant literature, 7 electronic databases including the Cochrane Library, PubMed, Excerpt Medica Database, Chinese Biomedical Literature Database, China National Knowledge Infrastructure, China Scientific Journal Database, and Wanfang Database will be systematically retrieved by 2 independent researchers from their inceptions to September 2020. Additionally, we will also search Google scholar, Baidu scholar, conference proceedings, clinical registration websites, and reference lists of associated reviews to identify grey literatures. An example of search strategy for PubMed database was as follows, and the similar search strategies will be utilized to other electronic databases:

#1 Search: Search: (“Liver Neoplasms”[Mesh]) OR (((((((((((((((((((((Neoplasms, Hepatic[Title/Abstract]) OR (Neoplasms, Liver[Title/Abstract])) OR (Liver Neoplasm[Title/Abstract])) OR (Neoplasm, Liver[Title/Abstract])) OR (Hepatic Neoplasms[Title/Abstract])) OR (Hepatic Neoplasm[Title/Abstract])) OR (Neoplasm, Hepatic[Title/Abstract])) OR (Cancer of Liver[Title/Abstract])) OR (Hepatocellular Cancer[Title/Abstract])) OR (Cancers, Hepatocellular[Title/Abstract])) OR (Hepatocellular Cancers[Title/Abstract])) OR (Hepatic Cancer[Title/Abstract])) OR (Cancer, Hepatic[Title/Abstract])) OR (Cancers, Hepatic[Title/Abstract])) OR (Hepatic Cancers[Title/Abstract])) OR (Liver Cancer[Title/Abstract])) OR (Cancer, Liver[Title/Abstract])) OR (Cancers, Liver[Title/Abstract])) OR (Liver Cancers[Title/Abstract])) OR (Cancer of the Liver[Title/Abstract])) OR (Cancer, Hepatocellular[Title/Abstract]))
#2 Search: (((((((*Brucea javanica* oil emulsion[Title/Abstract]) OR (BJOE[Title/Abstract])) OR (BJOEI[Title/Abstract])) OR (Javanica oil emulsion[Title/Abstract])) OR (Seed oil of *Brucea javanica*[Title/Abstract])) OR (Bruceae Fructus[Title/Abstract])) OR (Yadanzi[Title/Abstract])) OR (Ya-dan-zi[Title/Abstract])
#3 Search: (((((((((randomized controlled trial[Title/Abstract]) OR RCT[Title/Abstract]) OR random[Title/Abstract]) OR randomly[Title/Abstract]) OR random allocation[Title/Abstract]) OR allocation[Title/Abstract]) OR randomized control trial[Title/Abstract]) OR controlled clinical trial[Title/Abstract]) OR clinical trial[Title/Abstract]) OR clinical study[Title/Abstract]
#1 and #2 and #3

### 2.3 Study selection and data extraction

#### 2.3.1 Selection of studies

**Figure 1:**
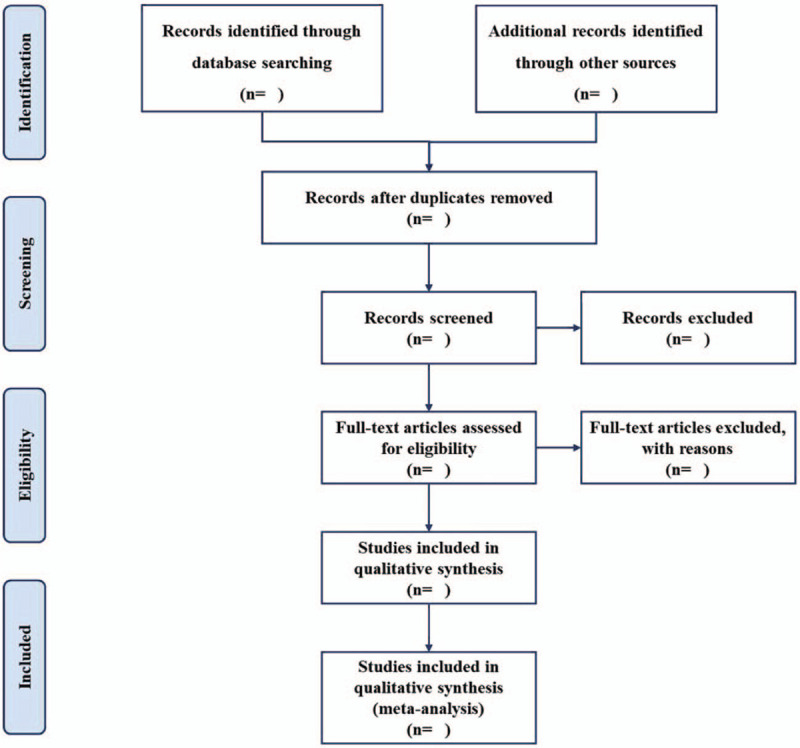
Flow chart of study selection.

The electronic citations extracted out from the above databases will be managed by Endnote X9 software (Clarivate Analytics, Philadelphia, USA).^[24]^ Any duplicates will be removed. Two independent researchers will review the titles/abstracts of all searched studies in accordance with the inclusion and exclusion criteria. Full papers of potential studies will be reviewed if necessary. Any disagreements generated between the 2 researchers will be solved by discussion with another researcher. All excluded studies will be listed in a table with reasons. A PRISMA flow chart (Fig. 1) will be drawn to present the whole process of study selection.

#### 2.3.2 Data extraction and management

Two researchers will extract relevant data independently with the standardized sheet recommended by the Cochrane Handbook of Systematic Reviews of Interventions. The data of those qualified articles will be export to Microsoft Excel, which includes basic information (registered identification, first author, author's unit, country, and publication year), research design (sample size, random sequence generation, allocation concealment, analysis of the data, processing of missing data, blinding of the participants, blinding of the outcome measurement, and blinding of the assessors), participants (disease, age, disease stage, and diagnostic criteria), details of treatment and comparison (eg, delivery methods, dosage, and frequency), outcomes (outcome measurement), adverse events, conflicts of interest, and other essential information. If unclear or missing data is examined, we will contact primary authors to achieve it whenever possible. If there is any dispute in the data extraction process, it will be submitted to a third researcher for processing. Once the extraction is complete, the 2 researchers will check with each other to ensure the accuracy of the data.

#### 2.3.3 Assessment of risk of bias

A tool introduced in the Cochrane Handbook for Systematic Reviews of Interventions will be used to assess a broad category of biases.^[25]^ This tool has 7 domains include random sequence generation, allocation concealment, blinding of the participants and personnel, blinding of the outcome assessments, incomplete outcome data, selective reporting, and other sources of bias. The assessment will be classified as “Low risk,” “High risk” or “Unclear risk.” Inconsistencies will be resolved by discussion within the group.

#### 2.3.4 Synthesis of data

RevMan 5.4 (The Cochrane Collaboration, Oxford, England) and Stata 16.0 (Stata Corporation, College Station, TX) will be applied to carry out statistical analysis. Risk ratio or odds ratio will be used for dichotomous outcomes. Mean difference or standardized mean difference will be used for continuous outcomes. The confidence intervals for both dichotomous and continuous variables will be set to 95%.

#### 2.3.5 Assessment of heterogeneity

Cochrane *X*^2^ and *I*^*2*^ tests will be conducted to assess the heterogeneity analysis between studies.^[26]^ If *P* ≥ .05 and *I*^*2*^ ≤ 50%, it suggests that no statistical heterogeneity is observed between subgroups, and the Mantel-Haenszel fixed model will be employed for meta-analysis. If *P* < .05 and *I*^*2*^ > 50%, it is considered that there is great heterogeneity between the studies, and the random effect model will be used.

#### 2.3.6 Subgroup analysis

If the necessary data are available in the case of high heterogeneity, we will conduct subgroup analysis according to the region of the studies, age, stage of the subjects, types of treatments, and different outcomes. The credibility of the subgroup analysis will be evaluated in term of the guidance.^[27]^ If there is a substantial heterogeneity and quantitative synthesis is not appropriate, the results will be presented in the form of tables and figures.

#### 2.3.7 Sensitivity analysis

Sensitivity analysis will be conducted to identify the stability and the robustness of the study results by removing low quality studies.

#### 2.3.8 Assessment of reporting bias

A funnel plot and Egger regression test will be used to identify the possible publication bias when more than 10 studies are included.^[28,29]^*P* < .05 is considered to have publication bias.

#### 2.3.9 Grading the quality of evidence

We will assess the quality of evidence using the The Grading of Recommendations Assessment, Development and Evaluation (GRADE), a widely used tool in evaluating the quality of assessment.^[30]^ The quality of evidence will be graded as high, moderate, low, and very low.

### 2.4 Patient and public involvement

Patient and public were not involved in this study.

### 2.5 Ethics and dissemination

This systematic review will not require ethical approval because there are no data used in our study that are linked to individual patient data. We will disseminate the results of this systematic review by publishing the manuscript in a peer-reviewed journal or presenting the findings at a relevant conference.

## 3 Discusssion

Liver cancer has become one of the main diseases threatening human health in the 21st century.^[31]^ BJOE, a famous Chinese patent medicine extracted from *Bruceae Fructus (Yadanzi)*, has been widely used for the treatment of liver cancer in clinical practice in China. However, no systematic review related to BJOE for liver cancer has been published currently. In this study, we will conduct systematic review and meta-analysis to provide more evidence on the effectiveness and safety for it. The findings of this studymay provide more guidance for clinicians in the treatment of liver cancer.

## 4 Amendments

If amendments are needed, we will update our protocol to include any changes in the whole process of research.

## Author contributions

**Conceptualization:** Didi Luo, Haiming Zhang.

**Data curation:** Didi Luo, Daorui Hou, Meiling Feng.

**Formal analysis:** Tiancheng Wen, Meiling Feng.

**Funding acquisition:** Haiming Zhang.

**Investigation:** Didi Luo, Daorui Hou, Tiancheng Wen.

**Methodology:** Didi Luo, Daorui Hou, Haiming Zhang.

**Project administration:** Haiming Zhang.

**Resources:** Didi Luo, Tiancheng Wen, Meiling Feng.

**Software:** Didi Luo, Daorui Hou, Meiling Feng.

**Supervision:** Haiming Zhang.

**Writing – original draft:** Didi Luo, Daorui Hou.

**Writing – review & editing:** Didi Luo, Daorui Hou, Tiancheng Wen, Meiling Feng, Haiming Zhang.
